# Supplementary material for: The relationship between plant-eating and hair evacuation in snow leopards (Panthera uncia)
Source: PLoS One. 2020 Jul 31;15(7):e0236635. doi: 10.1371/journal.pone.0236635 (PMC7394552; doi:10.1371/journal.pone.0236635)
Supplement: S1 File — This file includes scatter plot of hair and three variables (s-plant, b-plant, a-plant) and results of the GLM. (DOCX) [file pone.0236635.s002.docx]

**
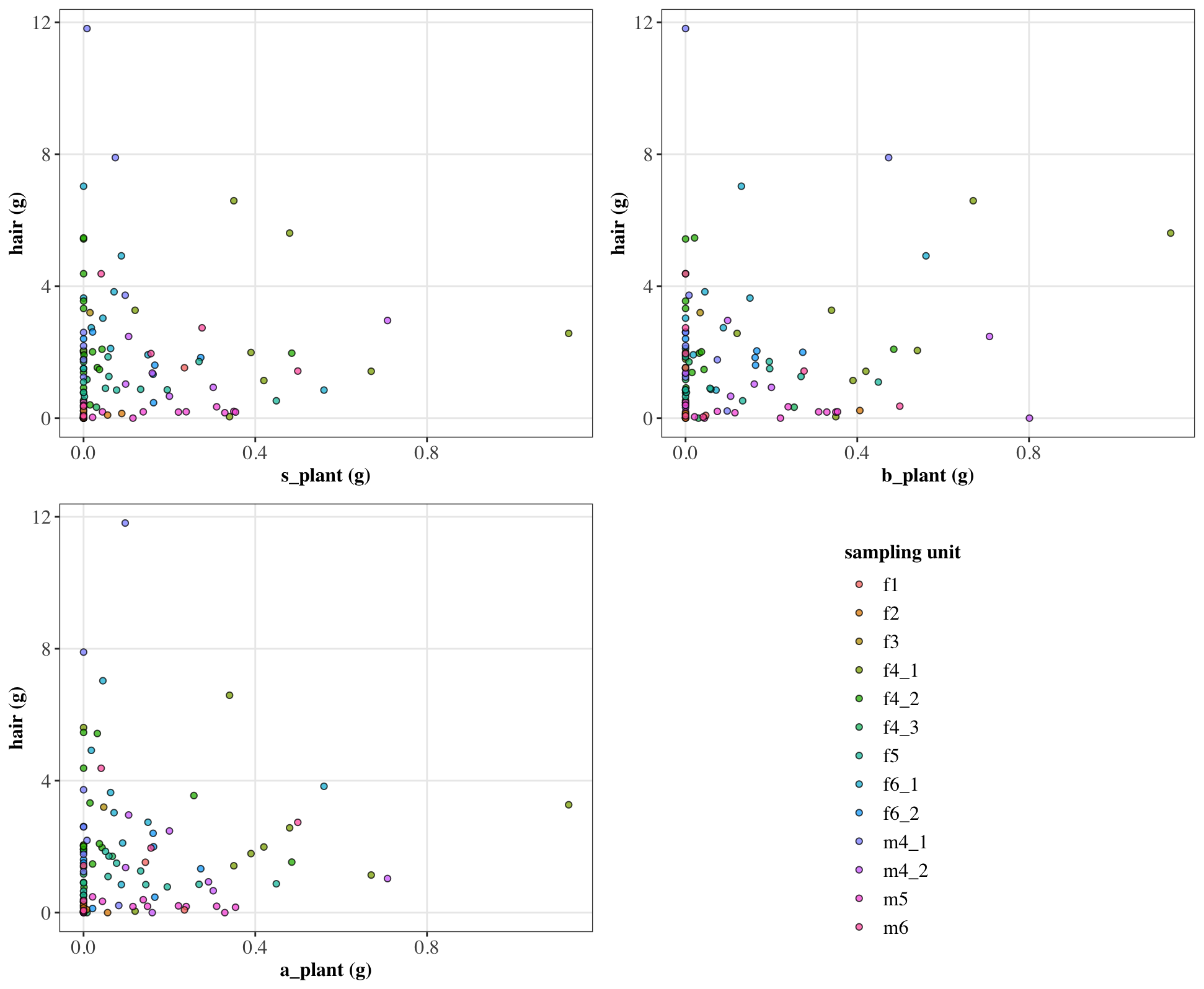
Figure. Scatter plot of objective variable and explanatory variables in Table 4.**

Different color represents difference of sampling unit (i.e. individual and sampling period).

**Table A. Estimated coefficients in each legend based on GLM (Bayesian estimation).** Coefficients are shown with [lower 95%CI, Higher 95%CI]. Estimates were not calculated for “f3” due to small sample size.

| Objective variable | hair | | |
| --- | --- | --- | --- |
| Explanatory variable | s-plant | b-plant | a-plant |
| Estimates |  |  |  |
| f1 | 2.3 [-18.7, 21] | -11.6 [106.9, 92.6] | -5.2 [-54, 48.1] |
| f2 | 3.3 [-100.7, 104.3] | 1.1 [10.2, 11.5] | 8 [-153.5, 167.9] |
| f3 | n.d. | n.d. | n.d. |
| f4_1 | 1.2 [-5.2, 8.8] | 2.7 [-2.8, 9.3] | 0.7 [-5.4, 8.7] |
| f4_2 | 0.2 [-4.4, 31.8] | 8.6 [-4.1, 35.5] | 7.4 [-4.7, 31.7] |
| f4_3 | 10.4 [-294, 320.9] | 1.1 [-35.9, 38.1] | 13.1[ -129, 145.5] |
| f5 | 0.1 [-2.7, 4.8] | 1.4 [-1.6, 6.0] | 0.3 [-2.4, 5.4] |
| f6_1 | -3.7 [-9.8, 7.8] | 7.8 [-3.4, 23.7] | 3.4 [-6.8, 20.3] |
| f6_2 | 1.4 [-10, 20.5] | 4.9 [-7.2, 24.6] | 1.3 [-10.8, 19.1] |
| m4_1 | 43.9 [-47.6, 148] | 7.4 [-9.9, 30.3] | 34.3[-41.5, 120.5] |
| m4_2 | 4.8 [-10.4, 19.1] | 0.2 [-10.9, 12] | 1.5 [-14.8, 19] |
| m5 | 0.9 [-3, 8.5] | 0.8 [-3, 8.8] | 1.4 [-2.7, 10.7] |
| m6 | 6.9 [-12.9, 27.2] | 0.8 [-15.5, 21.1] | 7.9 [-9.9, 27.9] |
