# Supplementary material for: The relationship between plant-eating and hair evacuation in snow leopards (Panthera uncia)
Source: PLoS One. 2020 Jul 31;15(7):e0236635. doi: 10.1371/journal.pone.0236635 (PMC7394552; doi:10.1371/journal.pone.0236635)
Supplement: Attachment — Submitted filename: REVIEW_SNOW_LEOPARDS_HMM_RESPONSE.pdf [file pone.0236635.s005.pdf]

Editorial Committee PLOS ONE

Thank you for considering my name for the revision of the manuscript: The relationship between plant-eating and hair evacuation in snow leopards (*Panthera uncia*) by Hiroto Yoshimura et al.

First at all I would like to congratulate the authors for this very valuable study on snow leopards feeding behavior, that will definitely add important elements to the understanding of the species ecology and provides management elements for both *in situ* and *ex situ* conservation scenarios.

In this paper the authors analyzed plant material and hair amount in scat samples of 13 (11) snow leopards (7 females, 6 males) kept in zoos in Japan, in order to test if plant consumption facilitates or it is associated with i) vomiting and ii) hair evacuation. The authors used a GLM to analyze their data and concluded that although plant consumption is common among snow leopards, there is no relationship between the ingestion of plant material and vomiting for hair evacuation. The manuscript is professionally written, and information is presented in a highly organized manner.

Due to the importance of the species as an icon of conservation and its condition as an endangered species, I recommend considering this research for editorial space in the journal.

However, several aspects need to be addressed by the authors prior publication. My major concerns are referred to the introductory and discussion sections. A greater effort needs to be made on contextualizing:

1) The threaten category of the species. I could not find a single comment on major threats facing by the species in the wild not even the UICN category was cited in the text. The authors, major players on *in situ* conservation efforts, should include in an explicit way, recommendations that contribute to the management of the species in captivity derived from the results of the present study. This also works for *ex situ* conservation efforts. The discussion section would be enriched by a comment of the authors on the applicability of results of this research in wildlife management.
2) On the same line of ideas, it would be great for the authors to share their appreciation on the importance of conducting research taking advantage of captive animals at zoos.

Although the authors make a great effort in providing information on the existing hypotheses on carnivore plant consumption, little information is provided on what kind of determinants are potentially related with plant consumption by snow leopards in their natural environments.

I fully understand that, as in many other wild felid species, the collection of information on diet for *Panthera uncia* is difficult in the wild. I highly recommend the authors to include some information on the general climate determinants across snow leopards´ distribution, to account for both plant phenology and animal prey availability throughout the year. It would also be important to know: i) if plants found in snow leopards scats are common or not in the wild; ii) if they coexist with animal preys used by the cats, or if the cats must move to other areas to get them; and finally, iii) what we know about the pattern of spatial distribution of these plants (aggregated, scattered, other), including a brief description on their elevational limits. These comments are relevant since Wegge et al. (2012) reported the presence of only plants in scats of snow leopards; work cited by the authors.

Wegge P, Shrestha R, Flagstad O. Snow leopard Panther uncia predation on livestock and 359 wild prey in a mountain valley in Northern Nepal: implications for conservation management. 360 Wildlife Biol. 2012;18(2):131–41.

Besides these general aspects, it would be highly appreciated if the authors will address this punctual aspects:

- Since there are several and quite different evolutionary paths among felids, the lack of sugar testing in domestic cats is an ambiguous indication on the lack of importance of plants as a nutritious resource for felids. Independently of tasting sugar, plant resources could be selected for their use based on another type of clues such as olfactory clues.
- Line 97: replace the word calve, by cubs.
- Line 334, capital letter for the name of the genus in *Panthera unica*.
- AGE OF THE ANIMALS: There is not information on animals age, if it is not possible to determining age, please explain why it was not taking into account?
- EXCLUSION OF WOOD MATTER: The explanation on why wood was not included as plant material can be enriched, particularly if one of the hypothesis mentioned in the introductory section claims physical properties of fibers from plants help the elimination of hair material through the digestive tract.
- Eliminate the word “of”, and the plural in the word amount, on line 210: The scatter plot shows the relationship between of the amount of hair and plant included in scat…
- Authors mention: This indicates that plant-eating behavior is common in captive snow leopards and appears even if they are not starving… First, I consider that the relationship established is not causal, lines 250.251. Based on the text, none of the animals were observed under starving conditions
